# Kinetic properties of glucose 6-phosphate dehydrogenase and inhibition effects of several metal ions on enzymatic activity in vitro and cells

**DOI:** 10.1038/s41598-024-56503-6

**Published:** 2024-03-09

**Authors:** Lindan Sun, Binbin Sun, Yulei Zhang, Keping Chen

**Affiliations:** 1 School of Life Sciences, Jiangsu University, Zhenjiang, 212000 Jiangsu China; 2 Guangdong South China Sea Key Laboratory of Aquaculture for Aquatic Economic Animals, Guangdong Ocean University, Zhanjiang, China

**Keywords:** CCO, Glucose 6-phosphate dehydrogenase, Kinetic behavior, Metal ions, Yellow catfish, Biochemistry, Environmental sciences

## Abstract

Due to the non-degradable and persistent nature of metal ions in the environment, they are released into water bodies, where they accumulate in fish. In order to assess pollution in fish, the enzyme, glucose 6-phosphate dehydrogenase (G6PD), has been employed as a biomarker due to sensitivity to various ions. This study investigates the kinetic properties of the G6PD enzyme in yellow catfish (*Pelteobagrus fulvidraco*), and analyzes the effects of these metal ions on the G6PD enzyme activity in the ovarian cell line (CCO) of channel catfish (*Ictalurus punctatus*). IC_50_ values and inhibition types of G6PD were determined in the metal ions Cu^2+^, Al^3+^, Zn^2+^, and Cd^2+^. While, the inhibition types of Cu^2+^ and Al^3+^ were the competitive inhibition, Zn^2+^ and Cd^2+^ were the linear mixed noncompetitive and linear mixed competitive, respectively. In vitro experiments revealed an inverse correlation between G6PD activity and metal ion concentration, mRNA levels and enzyme activity of G6PD increased at the lower metal ion concentration and decreased at the higher concentration. Our findings suggest that metal ions pose a significant threat to G6PD activity even at low concentrations, potentially playing a crucial role in the toxicity mechanism of metal ion pollution. This information contributes to the development of a biomonitoring tool for assessing metal ion contamination in aquatic species.

## Introduction

All living organisms depend on metals as essential components, fulfilling three primary functions: providing structural support, acting as enzyme cofactors, and facilitating electron transport^1^. However, elevated concentrations of metal ions, including low levels of non-essential heavy metals such as Cadmium (Cd), can lead to significant health concerns^2^. Due to their non-degradable and persistent nature in the environment, metal ions accumulate in aquatic organisms, posing a potential risk to human health as they undergo biological amplification through the food chain^3,4^. As demonstrated in a previous study, heavy metal pollution in aquatic species has reached critical levels, with the accumulation of metals in the tissues of yellow perch serving as a reflection of local contamination levels^5^. Furthermore, Chan et al. reported the presence of metal ions in the gills, intestines, and muscles of aquatic organisms in a South China region affected by acid mine drainage^6^. The impact of heavy metals on fish biochemical parameters, histopathology in various vital organs, and molecular-level DNA alterations are of considerable significance^7^.

In response to metal ion pollution, identifying contamination markers represents a direct and practical approach to monitoring the safety of aquatic products. The impact of pollutants can be assessed through biochemical reactions, specifically biological or molecular biomarkers^8^. Biomarkers, encompass physiological and behavioral indicators, respond to exposure to contaminants and their effects^9^. Fish hematological characteristics are also commonly recognized as a highly suitable experimental model for various biotests and toxicity experiments^10^. The enzyme Glucose 6-phosphate dehydrogenase (G6PD, E.C 1.1.1.49), a rate-limiting enzyme, plays a central role in generating ribose and the reducing agent nicotinamide adenine dinucleotide phosphate (NADPH) through the pentose phosphate pathway (PPP)^11^. G6PD as a biomarker has been applied in various fields, such as hepatocellular carcinoma and Merkel cell carcinoma prognostic^12,13^. In the presence of NADP, G6PD catalyzes the conversion of glucose 6-phosphate into 6-phosphogluconate, which has been utilized as a biomarker for pollution-induced carcinogenesis in fish^14^. G6PD can also stimulate the activation of cytoplasmic nicotinamide adenine dinucleotide kinase (NADK1), producing NADP^+^ and contributing to the growth of NADPH and NADP^+^ pools under various oxidative stress conditions^15^. Previous research has demonstrated the utility of G6PD from earthworm species (*Eisenia fetida*) for monitoring metal ion contamination in soil ecology^16^. It has also been observed that certain metal ions can inhibit the activity of G6PD in Capoeta umbla liver and gill tissues^17^. In a prior study, heavy metal contamination levels were monitored by evaluating immunocytological and cytogenotoxic biomarkers in the gill and haemocyte tissues of green-lipped mussels (*Perna canaliculus*) in coastal waters^18^. However, the mechanism underlying the use of G6PD from yellow catfish (*Pelteobagrus fulvidraco*) for determining the extent of metal ion contamination remains unknown. The enzyme kinetics of G6PD from rainbow trout erythrocytes and grass carp hepatopancreas have been studied on numerous heavy metal ions (Ag^+^, Zn^2+^, Cd^2+^, and Cu^2+^)^19,20^.

Yellow catfish (*Pelteobagrus fulvidraco*) can be used as a species to monitor environmental pollution because of its living environment, wide distribution, sensitivity to various metal ions and the interaction between immune dynamics and environment^21^. This study delved into the inhibitory effects of heavy metals on G6PD activity both in vitro and cells. In vitro, G6PD was directly extracted from yellow catfish (*Pelteobagrus fulvidraco*) using affinity chromatography. Subsequently, the study examined G6PD enzyme kinetics and the impact of heavy metals on its enzymatic activity. As yellow catfish lack a cell line, channel catfish (Ictalurus punctatus) ovary (CCO) cells were employed to investigate enzyme activity in a cellular context, given the taxonomic similarity of channel catfish and yellow catfish as both belong to the Siluriformes group. Notably, this research probed the inhibitory effects of various metal ions (Cu^2+^, Al^3+^, Zn^2+^, and Cd^2+^) on enzymatic activity in vitro and within cells. The overarching goal was to provide comprehensive data to support the development of pollution guidelines for aquatic products, specifically focusing on the G6PD enzyme. Furthermore, the study also explored the potential therapeutic implications of G6PD as a target for intervention.

## Results

### G6PD kinetics properties from yellow catfish liver

**Figure 1:**
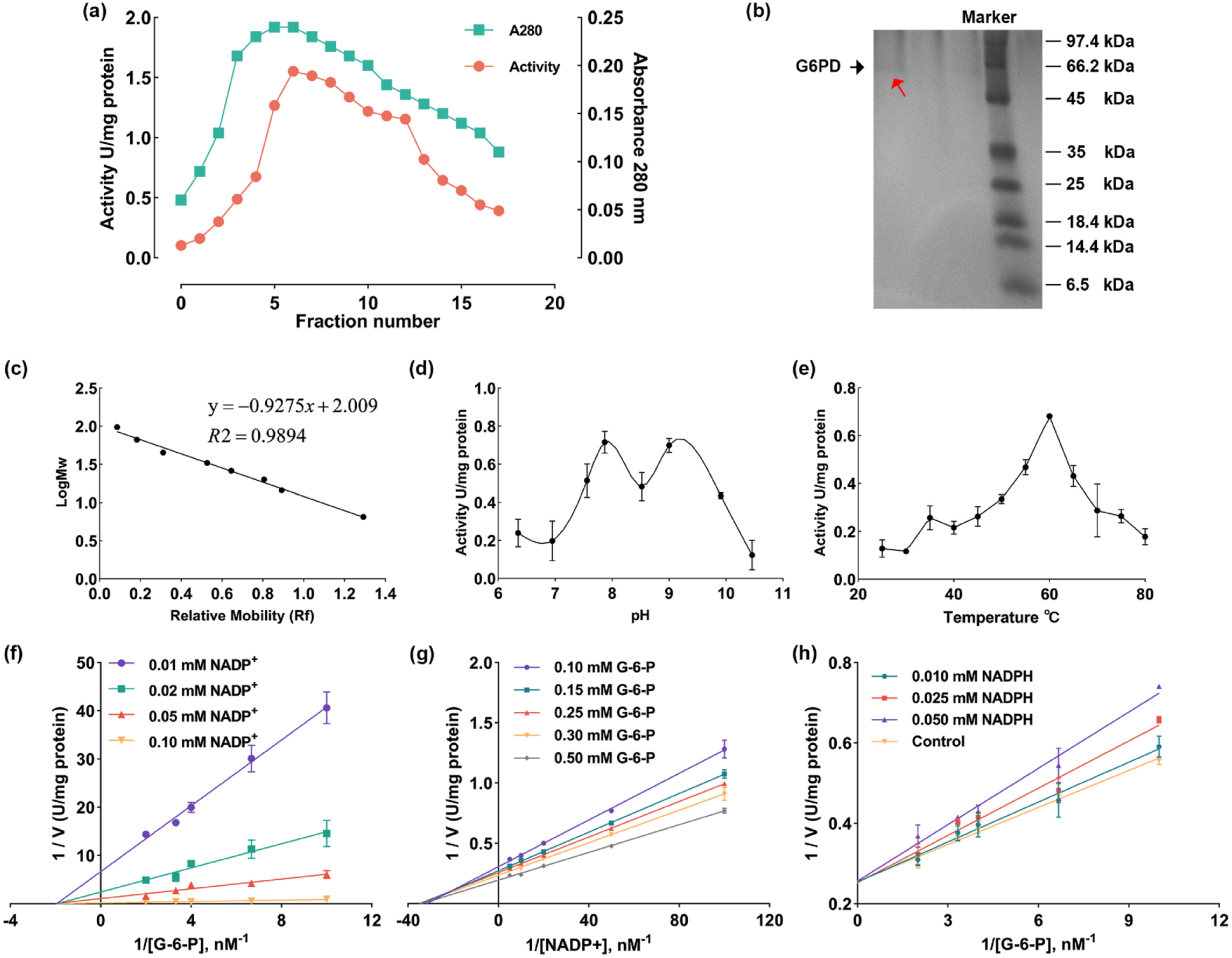
Purification and kinetic properties of G6PD isolated from yellow catfish liver. (**a**) Affinity column elution profile of G6PD. (**b**) SDS-PAGE photograph of G6PD. Lane 1: purified G6PD (red arrow). Lane 4: standard proteins. (**c**) G6PD standard molecule weighs Rf-logMw graph. (**d**) Effect of different pH on G6PD activity. (**e**) Effect of different temperatures on G6PD activity. (**f**) The double-reciprocal plot of initial velocity against G-6-P as varied substrate at different fixed NADP^+^ concentrations for the reaction catalyzed by G6PD from yellow catfish liver. (**g**) The double-reciprocal plot of initial velocity against NADP^+^ as varied substrate at different fixed G-6-P concentrations. (**h**) The double-reciprocal plots of the inhibition of G6PD by NADPH at three different concentrations to determine K_i_. The controls show reactions with no inhibitor present.

G6PD was eluted using buffer A supplemented with 0.177 mM NADP^+^ (Fig. 1a). After the purification process, the enzyme exhibited a specific activity of 0.83 U/mg of proteins, resulting in a notable 233.72-fold enhancement in purity (Table 1). The overall yield of the purification process reached approximately 58.24%. Furthermore, the purified G6PD presented a single band on the SDS-PAGE gel (Fig. 1b), with a molecular weight (Mr) measured at 68.89 kDa (Fig. 1c). A summary of the purification scheme for G6PD from yellow catfish liver can be found in Table 1.

**Table 1:** Purification scheme of G6PD from yellow catfish liver.

Purification step	Total volume (ml)	Activity (U/ml)	Total activity (U)	Protein (mg/ml)	Specific activity (U/mg)	Yield (%)	Purification (fold)
Homogenate	24.750	0.014	0.347	3.897	0.004	100.000	1.000
Supernatant	18.750	0.014	0.263	1.847	0.007	73.131	2.065
2ʹ,5ʹ-ADP-Sepharose 4B	9.500	0.021	0.200	0.025	0.831	58.243	233.718

With the increase in pH value, the activity of G6PD increased, and the maximum activity was 0.715 U/mg at pH 7.87. At pH 8.52, the activity thereafter declined. Remarkably, the activity of G6PD increased once more and reached the maximum value of 0.665 U/mg at pH 9.0. Next, as the pH value increased, the enzyme activity reduced (Fig. 1d). In the determination of optimum temperature, the activity of G6PD increased as the temperature rose, peaking at 0.682 U/mg at 60 °C. After that, the activity of G6PD declined as the temperature rose (Fig. 1e).

Figure 1f illustrates the Lineweaver–Burk double-reciprocal plots for G-6-P as the substrate at different NADP^+^ concentrations. While, under the same G-6-P concentration, with the NADP^+^ concentration increased, the values of 1/V were decreased, which represented that the maximum Vmax of G6PD appeared in the 0.1 mM NADP^+^. In contrast, Fig. 1g presents similar plots for NADP^+^ as the substrate at various G-6-P concentrations. The maximum Vmax of G6PD appeared in the 0.5 mM G-6-P under the same NADP^+^ concentration. In the results, the points where these lines intersected the horizontal axis indicating a sequential mechanism for the catalytic process facilitated by G6PD in yellow catfish liver.

To investigate how NADPH inhibits G6PD, we employed G-6-P as a substrate and generated Lineweaver–Burk double-reciprocal plots at different NADPH concentrations, as depicted in Fig. 1h. The graph revealed that NADPH competes with 6PGD. The determined Km values for G-6-P and NADP^+^ were 0.479 mM and 0.029 mM, respectively, while the V_max_ value was 2.83 U/ml (Table 2). The dissociation constant K_i_ for NADPH was calculated to be 0.092 mM.

**Table 2:** Kinetic parameters of G6PD from yellow catfish liver.

Parameters	Values
K_mG-6-P_ (mM)	0.479 ± 0.013
K_mNADP+_ (mM)	0.029 ± 0.002
V_max_ (U/ml)	2.830 ± 0.130
K_iNADPH_ (mM)	0.092 ± 0.001

### In vitro inhibition assays

**Figure 2:**
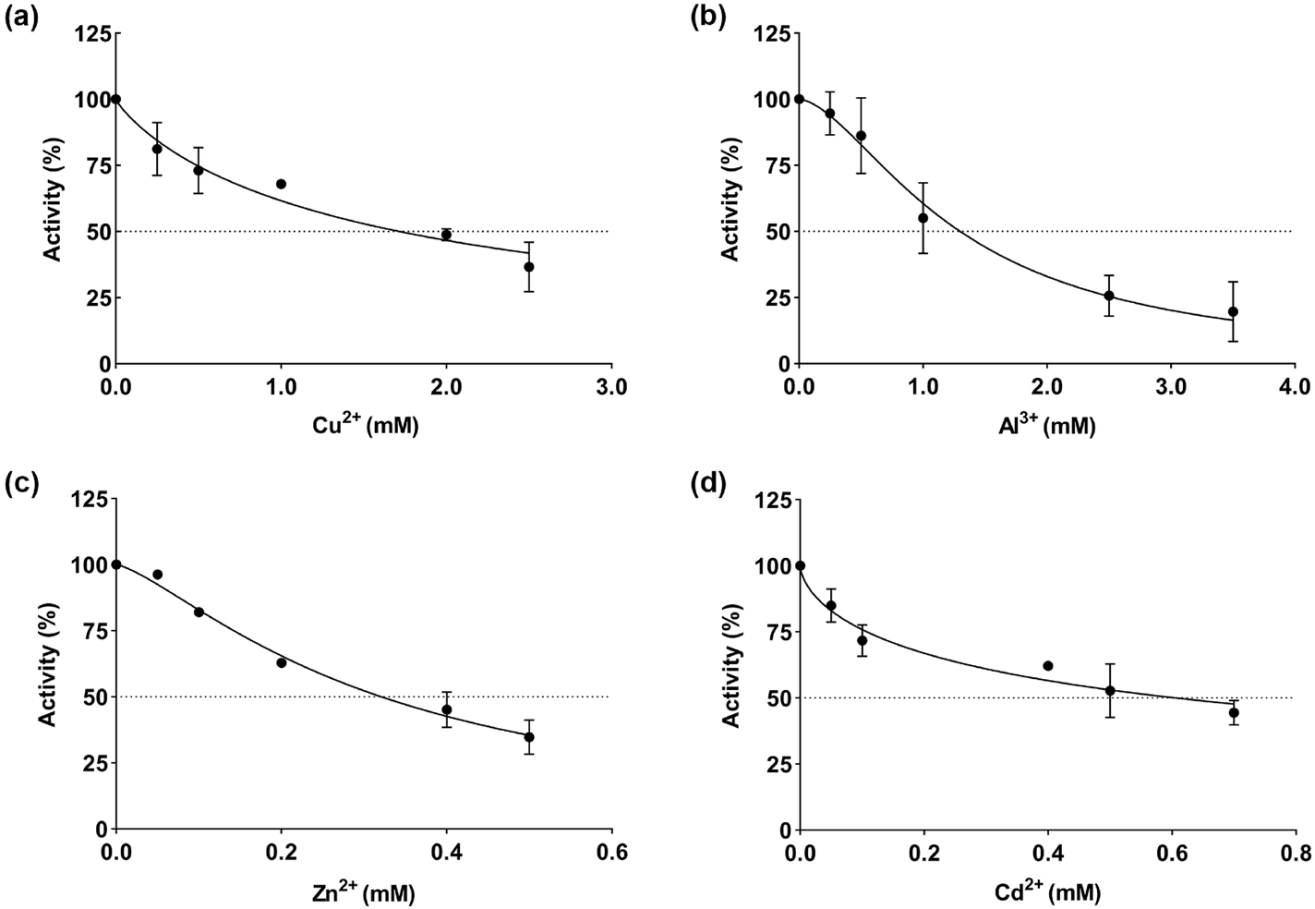
Activity (%) vs metals regression analysis graphs for yellow catfish G6PD in the presence of different metals concentrations (**a**) Cu^2+^, (**b**) Al^3+^, (**c**) Zn^2+^, (**d**) Cd^2+^.

**Figure 3:**
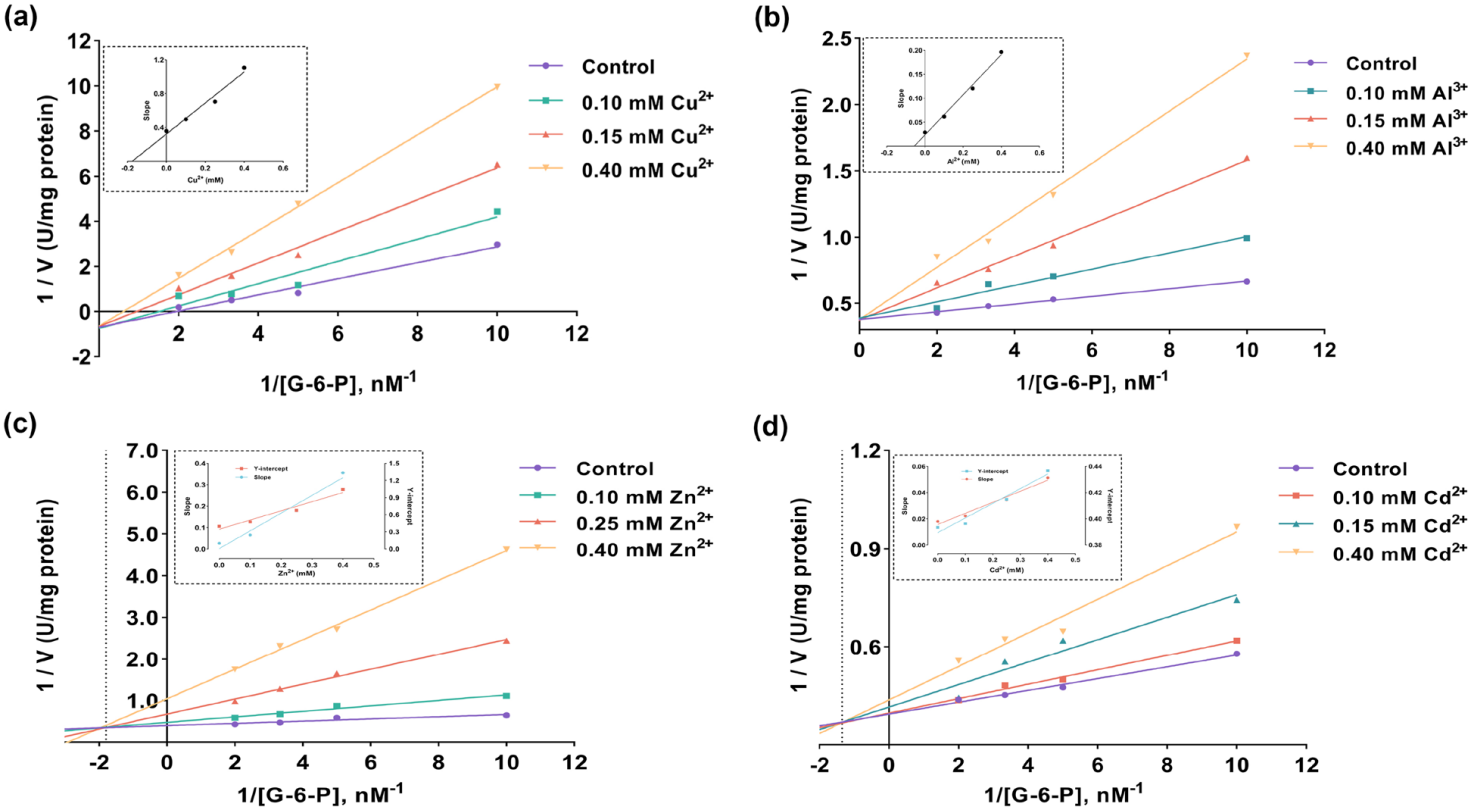
Double-reciprocal plots of the inhibition of 6PGD in yellow catfish by (**a**) Cu^2+^, (**b**) Al^3+^, (**c**) Zn^2+^, (**d**) Cd^2+^ at three different concentrations for determination of K_i_. The controls show reactions with no inhibitor present.

The regression analysis graphs for yellow catfish G6PD, depicting Activity (%) versus metals, are presented in Fig. 2. Based on the data, we calculated the IC_50_ values for Cu^2+^, Al^3+^, Zn^2+^, and Cd^2+^ to be 1.718 mM, 1.299 mM, 0.321 mM, and 0.606 mM, respectively (Fig. 2a–d, Table 3). The K_i_ constants for Cu^2+^ and Al^3+^ were determined through Lineweaver–Burk plots, resulting in values of 0.175 mM and 0.056 mM, respectively. Both Cu^2+^ and Al^3+^ exhibited competitive inhibition (Fig. 3a,b).

**Table 3:** In vitro the parameters which metal ions effect G-6-P.

Metal ions	IC_50_ (mM)	K_i_ (mM)	Inhibition type
		K_is_ K_ii_	
Cu^2+^	1.718	0.175	Competitive
Al^3+^	1.299	0.056	Competitive
Zn^2+^	0.321	0.002 0.216	Linear mixed noncompetitive
Cd^2+^	0.606	0.186 3.466	Linear mixed competitive

In the case of Zn^2+^ and Cd^2+^, the K_i_ constants indicated mixed-type inhibition patterns. Replots of the slope (K_is_) and intercept (K_ii_) from the double-reciprocal plots with inhibitor concentration yielded linear plots, suggesting a linear mixed-type inhibition. For Zn^2+^, the K_is_ was calculated to be 0.002 mM, and the K_ii_ was 0.216 mM. For Cd^2+^, the K_is_ was 0.186 mM, and the K_ii_ was 3.466 mM (Fig. 3c,d).

### In cells inhibition assays

**Figure 4:**
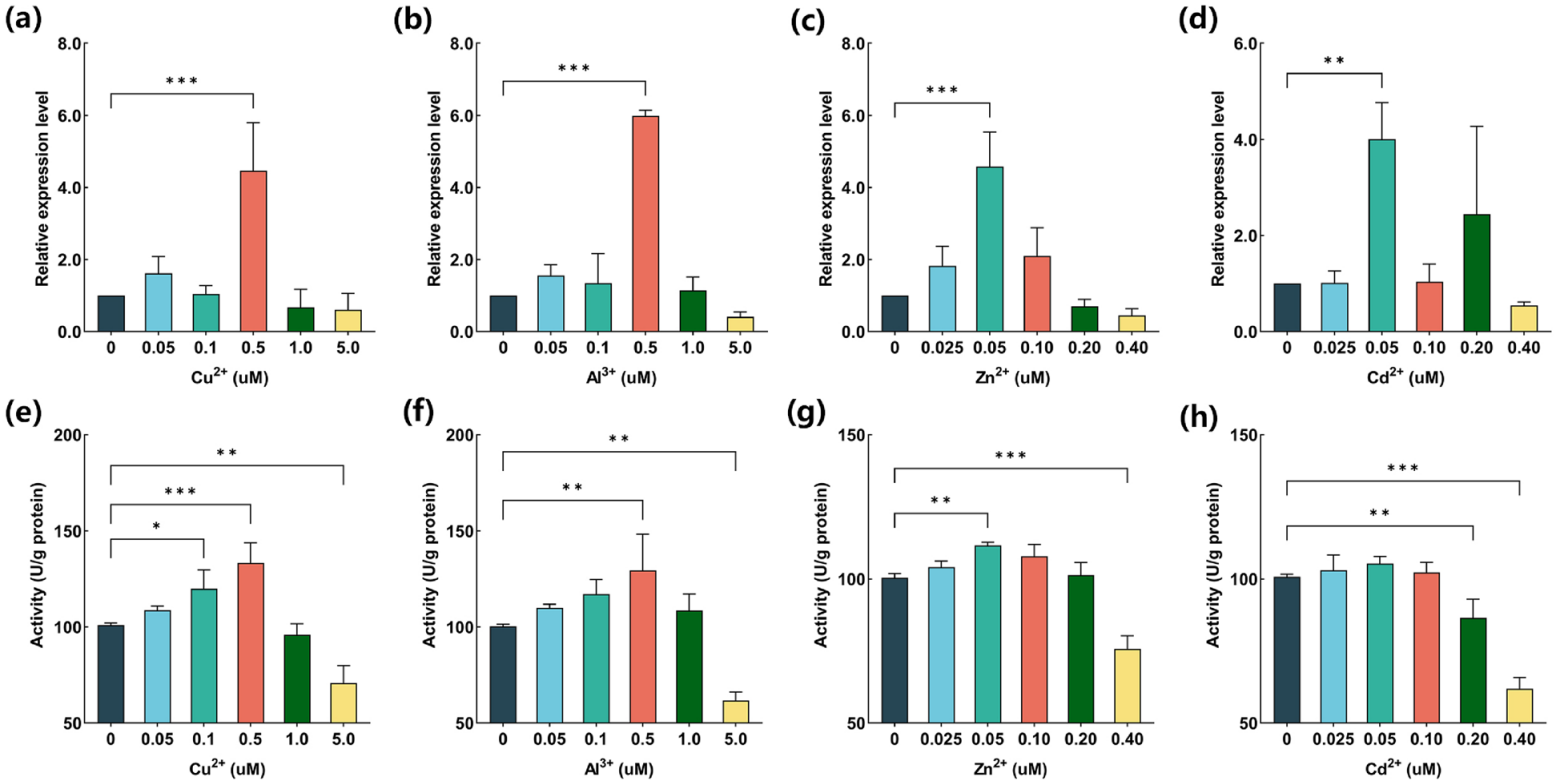
The effect of metal ion on mRNA and activity of G6PD in CCO by QRT-PCR in the presence of different metals concentrations. mRNA of G6PD on (**a**) Cu^2+^, (**b**) Al^3+^, (**c**) Zn^2+^, (**d**) Cd^2+^. Activity of G6PD on (**e**) Cu^2+^, (**f**) Al^3+^, (**g**) Zn^2+^, (**h**) Cd^2+^. The *, **, and *** represent significant differences with *p* < 0.05, < 0.01, and < 0.001.

Figure 4 demonstrate that for G6PD mRNA expression levels and enzyme activity under the four metal ions various concentration. The highest expression levels of G6PD mRNA were observed in the 0.5 μM group for Cu^2+^ and Al^3+^ (*p* < 0.001), and the lowest expression levels of G6PD mRNA were achieved in the 5.0 μM (Fig. 4a,b). Under the 0.05 μM Zn^2+^ condiation, the highest levels of G6PD mRNA were achieved (*p* < 0.001), while the similar results were observed from Cd^2+^ (*p* < 0.01). The lowest expression levels of G6PD mRNA were achieved at 0.4 μM in both Zn^2+^ and Cd^2+^ groups. This suggests that G6PD is more sensitive to Zn^2+^ and Cd^2+^ compared to Cu^2+^ and Al^3+^. Interesting, the change intention of four metal ions impact on enzyme activity of G6PD were similar to the expression levels of G6PD mRNA. Among Cu^2+^, Al^3+^, and Zn^2+^, the highest enzyme activity of G6PD were achieved that were correspondence to the G6PD mRNA expression (*p* < 0.01) (Fig. 4e–g). The Cd^2+^ group had conversed result, the G6PD activity were similar under the 0.025, 0.05, and 0.1 μM and decreased at 0.2 and 0.4 μM (*p* < 0.01) (Fig. 4 h). Additionally, it is noteworthy that both G6PD mRNA and activity exhibited an initial increase with the rise in metal concentration, reaching peak levels, then declining as the metal concentration further increased. This indicates that lower metal concentrations promote enzyme activity, while higher metal concentrations inhibit enzyme activity.

### Metal ions with G6PD molecule docking model

**Figure 5:**
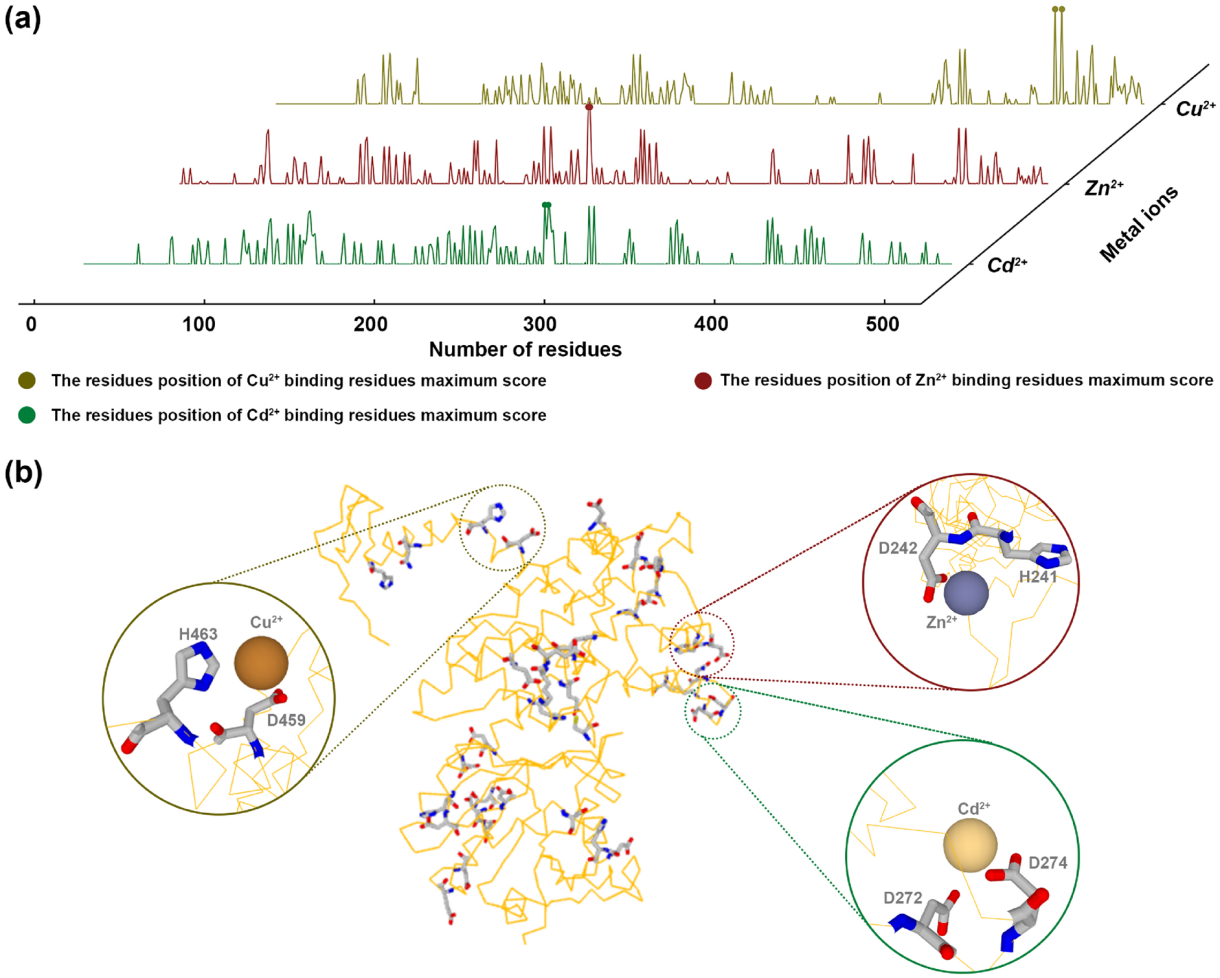
Cu^2+^, Zn^2+^, and Cd^2+^ docking with G6PD. Score diagram of binding residues of metal ions in G6PD amino acid sequences (**a**). Schematic diagram of the interaction between Cu^2+^, Zn^2+^, and Cd^2+^ and amino acids at the highest score binding residues position (**b**).

In order to better deeply understand the molecular mechanism of metal ions inhibit the G6PD. The metal ions dock G6PD molecular docking model was built using the online Metal Ion-Binding site prediction and modeling server (http://bioinfo.cmu.edu.tw/MIB2/)^22^. Among the test four metal ions, Cu^2+^, Zn^2+^, and Cd^2+^ were predicted due to the limitation of the server. The primary accession number for the G6PD sequences, which were acquired from Uniprot, is F1Q883. The maximum score of Cu^2+^ for the binding residues was 7.523, as expected by the data; Cu^2+^ may have interacted with aspartic acid at position 459 and histidine at position 463 (Fig. 5a,b, Supplementary Materials Table S1). Likewise, Zn^2+^ and Cd^2+^ had the highest scores of 6.020 and 4.519, respectively (Supplementary Materials Table S1). While aspartic acid and histidine will interact with the Zn^2+^ at positions 242 and 241. Then the Cd^2+^ interacts with aspartic acid at positions 272 and 274 (Fig. 5a,b).

## Discussion

A surge in environmental pollution has accompanied the rapid expansion of urbanization and industrialization^23^. Earlier studies have reported that heavy metal concentrations, particularly for elements such as Hg and Co, were elevated in the groundwater of urban areas compared to non-urbanized regions within the Pearl River Delta^24^. Additionally, heavy metals were detected in the tissues of aquatic organisms in areas affected by acid mine drainage in southern China^6^. The persistent and non-degradable nature of metal ions in the environment makes them readily available to aquatic species. This, coupled with their potential for biological amplification within the food chain, raises concerns regarding the potential risks to human health associated with metal ion exposure^3,4^.

In this study, we aimed to assess the impact of metal ions on G6PD to contribute to developing a biomonitoring tool for evaluating metal ion contamination in aquatic species and establishing standards. The purification of G6PD from yellow catfish liver was achieved through ultracentrifugation and 2ʹ,5ʹ-Sepharose-4B affinity chromatography effectively separated G6PD from 6PGD^25^. Several organisms have been shown to use similar purification processes for G6PD^26,27^, due to the highest yield, specific activity, and cost-effective purification technique^28^. The specific activity of G6PD obtained in our study was measured at 0.83 U/mg protein, and difference from previous reports^17,29^. The variations in specific activities of G6PD could be attributed to the enzyme's different sources and origins^23^. Likewise, the G6PD exhibited a molecular weight of 68.89 kDa, which may have two maximum activity values according to the LOWSS linear regression analysis. It is plausible that the G6PD active site harbors multiple ionizable groups^30^. Additionally, at pH 7.87 and 9.0, the hydrogen ion from the –COOH group may transfer to the –NH_2_ group, promoting the formation of bonds between the enzyme and substrate and increasing G6PD activity^31^. Nevertheless, this work did not examine the structural makeup of G6PD from yellow catfish. On the other hand, G6PD may be more thoroughly analyzed using enzymatic fingerprinting, a potential substitute and complement to nuclear magnetic resonance spectroscopy to fully comprehend the impact of the structural composition of G6PD on enzyme activity^32^.

The kinetic mechanism of G6PD in our study followed an 'Ordered Bi' sequential mechanism, consistent with findings in other animal species^33^. Notably, the K_m_ values for G6PD and NADP^+^ that we determined in our research were lower than those reported in rainbow trout^34^ but higher than the values observed in *Aspergillus niger*^35^. Our study revealed that the K_m_ for G6PD was higher than that for NADP^+^, suggesting that 6PGD has a lower affinity for G6PD compared to NADP^+^^33^. This finding contrasts with previous reports where the Km for NADP^+^ was higher than G6PD^36,37^. Additionally, our investigation showed that NADPH competitively inhibited G-6-P, with a K_i_ of 0.092 mM. Similar results were observed in Thermotoga maritime (0.11 mM)^38^, but these values were higher than those reported in certain microorganisms (where NADPH exhibited competitive inhibition with a K_i_ of 0.012 mM)^27,35,39^. Furthermore, we found that NADPH inhibited G6PD in a noncompetitive manner, with a K_i_ constant of 0.144 mM^40^. These findings emphasize the metabolic control of G6PD enzyme activity by the cytosolic ratio of free NADP^+^ to NADPH^41^.

In our study, we observed a decrease in G6PD enzyme activity with an increase in the concentration of metal ions. This trend is consistent with findings from G6PD isolated from grass carp^30^. Our results indicated that G6PD is most sensitive to Zn^2+^ among the tested metal ions in vitro. Based on semi-empirical and qualitative theories, such as Parr and Pearson's soft acid–base (HSAB) principle, the selectivity and specificity of G6PD may have changed^42,43^. Among the metal ions have the ability to establish robust interactions with protein –SH, –NH, and –COOH groups^44^, and the metals can form covalent unions with –SH groups^45^, acting as a soft Lewis base and changing the structure and function of G6PD. Zn^2+^, Al^3+^, and Cu^2+^ from grass carp were comparable to those from our yellow catfish (Cu^2+^, Al^3+^, and Zn^2+^ IC_50_ values of 1.718 mM, 1.299 mM, and 0.321 mM, respectively)^30^. However, for Cd^2+^, a soft Lewis acid, the IC_50_ value was noticeably lower than grass carp. In terms of inhibition types, we determined that Cu^2+^ and Al^3+^ exhibited competitive inhibition, indicating that these metals compete with G-6-P for enzyme binding sites, adversely affecting the pentose phosphate pathway (PPP)^46^. However, the inhibition type for Cu^2+^ in grass carp was non-competitive^30^. Both Zn^2+^ and Cd^2+^ demonstrated a linear mixed-type inhibition pattern in our study. Zn^2+^ exhibited competitive inhibition but was different in grass carp^30,37^. These findings would suggest that there are structural variations between the G6PD of yellow catfish and grass carp and may be attributed to variations in dietary habits and growing environments, resulting in different physiological responses of the same enzyme to the same metal element in different species^47^.

The mRNA and enzyme activity data reveal that G6PD in CCO (channel catfish ovary cells) exhibits different sensitivities to metal ions compared to the isolated enzymes. The concentrations of heavy metals used in our study closely approximate those found in polluted waters, as referenced in various studies conducted in southeastern Nigeria^48^, China^49^, and land-based fish farms in Atlantic Canada^50^. In our experiments, both mRNA and enzyme activity levels of G6PD initially increased with the rise in metal ion concentration (ranging from 0 to 0.5 μM for Cu^2+^ and Al^3+^, and from 0 to 0.05 μM for Zn^2+^ and Cd^2+^). Zn^2+^ and Cd^2+^ share comparable chemical characteristics, are members of the IIB groups on the periodic table, and are found in metalloproteins in tetrahedral configurations^51^. This would account for why Zn^2+^ and Cd^2+^ have comparable activity in CCO and how they swap places in proteins without affecting G6PD function^52^, as well both cations may have the same Lewis acid–base capacity. However, the IB and IIIA groups are home to Cu^2+^ and Al^3+^, respectively^53^. The maximal activity of G6PD indicates that the protein responds to both cations similarly. The reason for the early G6PD maximum activity in Zn^2+^ and Cd^2+^ solution compared to Cu^2+^ and Al^3+^ could be that Zn^2+^ and Cd^2+^ have more active polarization in the carbonyl group^45^. Moreover, cells and organisms require trace amounts of Cu^2+^, Zn^2+^, and Al^3+^ for growth. Additionally, low concentrations of metal ions can stimulate stress responses in cells^54^ and may promote improved immune capabilities^55^. However, higher metal ion concentrations negatively impact the organism's immune system^56^.

Metal ions interact with G6PD via the molecular docking model can better understand the molecular mechanism of metal ions that inhibit G6PD^22^. According to our predicted results, Cu^2+^, Zn^2+^, and Cd^2+^ mainly interact with aspartic acid and histidine. Because histidine can be charged or neutral at physiological pH levels, it is particularly well-suited to take part in the reaction process^57^. Histidine can operate as an acid, alkali, or nucleophile and can also help to stabilize the reaction's intermediate state^58^. While aspartic acid's side chain is short, which contributes to its relative rigidity and fixed position, which aids in catalysis^58^. Cu^2+^, Zn^2+^, and Cd^2+^ are transition metal ions that interact with the enzyme's surface charge and influence the ionization of certain amino acid residues, and then the structure of the enzyme is altered^59^. Subsequently, there was a decrease in G6PD activity as the quantity of metal ions increased (ranging from 0.5 to 5.0 μM for Cu^2+^ and Al^3+^, and from 0.05 to 0.4 μM for Zn^2+^ and Cd^2+^). This could be attributed to the production of reactive singlet oxygen, which can damage DNA and amino acids^60^. Thus, the antioxidant defense was altered in the organisms^61^. Due to the limitation of our experimental, the more accurate molecular mechanism of the inhibition of G6PD by four metal ions should be further studied.

## Conclusion

In our study, we successfully isolated, purified, and characterized G6PD from yellow catfish liver. We also investigated the effects of metal ions on enzyme mRNA levels and enzyme activity both in vitro and in cells. When compared to other species, most of the parameters we examined, including specific activity, molecular weight, Km, Vm, and Ki, remained relatively consistent in yellow catfish, suggesting a common function in the properties of the protein. However, we did observe some differences, indicating a divergence between yellow catfish and other species. Based on our findings regarding the inhibitory effects of specific metal ions on G6PD, we hypothesize that if these metal elements exceed a certain threshold level, they could pose a hazard to fish and, ultimately, to human health due to consuming of contaminated aquatic species. This concern arises from the non-degradable and long-lasting nature of metal ions in the environment. In assessing metal ion contamination in aquatic species, it is crucial to consider the potential utility of G6PD as both a pollution biomarker and a therapeutic target. G6PD's role in responding to metal ion exposure highlights its significance in understanding and monitoring the impact of environmental pollution on aquatic ecosystems and food safety.

## Materials and methods

### Fish husbandry and sample collection

2ʹ,5ʹ-ADP-Sepharose 4B was procured from Pharmacia Fine Chemicals in Uppsala, Sweden, while protein markers were sourced from TIANGEN^®^. Other chemicals, including 2-mercaptoethanol (2-ME), NADP^+^, NADPH, Glucose-6-phosphate (G-6-P), and all additional reagents, were obtained from Sigma-Aldrich Chemical Co. in Missouri, USA. The CCO cells were cultured and maintained at 25 °C in MEM (minimal essential medium) supplemented with 10% FBS (GIBCO).

Liver samples, weighing 4 g per fish, were promptly isolated using sterile forceps on ice. A subset of 20 fish was selected for subsequent analysis.

### Purification and properties of liver G6PD

The purification of G6PD from yellow catfish liver was conducted following a previously published procedure^26,30,62^. The purification process comprised ultracentrifugation and 2ʹ,5ʹ-ADP-Sepharose 4B affinity chromatography and was carried out at a temperature of 4 °C.

Initially, the liver was sliced, and the samples were rinsed with ice-cold saline to eliminate residual blood. Subsequently, the samples were homogenized in a glass-Teflon homogenizer using three volumes of 10 mM Tris–HCl buffer (designated as buffer A), which included 1 mM EDTA and 5 mM 2-ME, and had a pH of 7.42. The homogenate was centrifuged at 100,000*g* for 60 min at 4 °C. The resulting supernatant was applied to a 2ʹ,5ʹ-ADP-Sepharose 4B column that had been pre-equilibrated with buffer A. The column was washed with 50 ml of buffer A, which continued until the final absorbance at 280 nm dropped below 0.01. Elution was performed using 30 ml of 10 mM Tris–HCl, 5 mM 2-ME, 1 mM EDTA, and 0.177 mM NADP, and had a pH of 7.42. Flow rates for both washing and equilibration were set at 16.2 ml/h. The purification scheme for G6PD from yellow catfish liver is depicted in Fig. 1A. Fractions 5–10 in Fig. 1A were combined and used as the analyzed sample.

The determination of G6PD activity was conducted at 25 °C by measuring the rate of NADP^+^ reduction at 340 nm, following the method described by Beutler^63^. Protein concentrations were determined using the Bradford method, with bovine serum albumin as the reference standard^64^. SDS-PAGE was carried out to assess enzyme purity and determine the apparent molecular mass of the subunit according to Laemmli's method^25,65^ and the SDS-PAGE raw image of G6PD was obtained by gel imaging instrument (GenoSens 1850). The standard proteins consist of rabbit phosphorylase b (97,400), bovine serum albumin (66,200), ovalbumin (45,000), lactate dehydrogenase (35,000), rease bap98l (25,000), beta-lactoglobulin (18,400), lysozyme (14,400), and bovine lung bacteriostatic enzyme (6500).

### The optimum pH and temperature of determination

The optimum temperature and pH of G6PD activity were determined, respectively. The optimal pH was carried on the 10 mM Tris–HCl, which has a pH range of 6.0–11.0. The 10 μl of the original enzyme was added to 90 μl of a pH-different Tris–HCl buffer for G6PD activity assay. At the optimum pH values, the determination of optimum temperature was carried on between 25 and 80 °C. The LOWESS approach was employed for the linear regression analysis, and the data was represented as the mean ± standard deviation (n = 3).

### Kinetic studies

To assess the G6PD kinetic parameters K_m_ and V_m_, Lineweaver–Burk plots were employed. These plots were generated using five different concentrations of NADP^+^ (0.01, 0.02, 0.05, and, 0.10 mM) while maintaining a constant concentration of G-6-P. Similarly, experiments were conducted with G-6-P at five different concentrations (0.10, 0.15, 0.25, 0.30, and 0.50 mM) with a fixed NADP^+^ concentration. All kinetic investigations were conducted at 25 °C and a pH of 7.87. The K_m_ and V_m_ values were deduced from the Lineweaver–Burk plots by analyzing the respective slopes and intercepts.

Various concentrations of NADPH (0.01, 0.025, and 0.05 mM) were employed to determine K_i_ values of G6PD pertaining to the inhibitor NADPH. G-6-P was used as a substrate at different concentrations (0.10, 0.15, 0.25, 0.30, and 0.50 mM, respectively). Activity measurements were conducted, and Lineweaver–Burk plots were constructed to determine K_i_ values and ascertain the inhibition type for NADPH^66^.

### In vitro effects of metal ions

To evaluate how different concentrated metal ions affect G6PD activity. Cu^2+^ (0, 0.25, 0.50, 1.00, 2.00, and 2.50 mM), Al^3+^ (0, 0.25, 0.50, 1.00, 2.50, and 3.50 mM), Zn^2+^ (0, 0.05, 0.10, 0.20, 0.40, and 0.50 mM), and Cd^2+^ (0, 0.05, 0.10, 0.40, 0.50, and 0.70 mM) were prepared using the salts of CuSO_4_, Al_2_(SO_4_)_3_, ZnSO_4_, and CdSO_4_ that had been diluted to 20 mM with 50 ml pure water. The control cuvette, devoid of any inhibitor, was used as a reference, and its activity was considered 100%. At each concentration, we performed triplicate assessments for each metal ion. An activity-inhibition graph was constructed for each inhibitor, and the concentrations of metal ions resulting in 50% inhibition (IC50) were determined from regression plots.

For each metal ion, we conducted experiments employing three distinct inhibitor doses (0.1, 0.15, and 0.4 mM for Cu^2+^, Al^3+^, Zn^2+^, and Cd^2+^, respectively) to determine K_i_ values. In these experiments, four concentrations of G-6-P were employed as substrates (0.10, 0.20, 0.30, and 0.50 mM, respectively). All experiments were repeated three times. Lineweaver–Burk plots were generated by comparing 1/V to 1/[S] data. These plots were used to ascertain the type of inhibition and calculate the K_i_ constant for each metal ion^66^.

### In cells effects of metal ions

To investigate the impact of metal ions on G6PD mRNA and activity in cells, we employed the concentrations that previous studies had reported of Cu^2+^ and Al^3+^ (0, 0.05, 0.1, 0.5, 1.0, 5.0 μM), Zn^2+^ and Cd^2+^ (0, 0.025, 0.05, 0.1, 0.2, 0.4 μM)^30^. The cells were cultured in MEM with 10% FBS for 12 h, after which they were washed twice with PBS and then exposed to varying concentrations of the respective metal ions (Cu^2+^, Al^3+^, Zn^2+^, and Cd^2+^) for a 2-h.

The mRNA levels of G6PD were determined via quantitative real-time PCR (qRT-PCR). The relative expression ratio was calculated using the 2^−ΔΔCT^ method, and all data were presented regarding relative mRNA expression. G6PD activity was assessed using a G6PD enzyme activity assay kit (Beyotime Biotechnology, S0189). The activity of the control cuvette, without any inhibitor, was set as 100%. These experiments were conducted in triplicate. Specific primer sequences can be found in Table 4

**Table 4:** Primer sequences used in the qRT-PCR experiment.

Gene name	Forward primer (5ʹ–3ʹ)	Reverse primer (5ʹ–3ʹ)
Glucose 6-phosphate dehydrogenase (G6PD)	CTGAGAAACCACCCCCTGTG	ACCTCCATTTGTCCGCTTGA
β-Actin	CACTGTGCCCATCTACGAG	CCATCTCCTGCTCGAAGTC

.

### Molecular docking model

Molecular docking model was established using the online Metal Ion-Binding site prediction and modeling server (http://bioinfo.cmu.edu.tw/MIB2/) (version: MIB2). The G6PD sequences were downloaded from Uniprot (https://www.uniprot.org/) (Primary accession: F1Q883).

### Statistical analysis

In this case, data are presented as the mean ± standard deviation (SD) of at least three replicates. In the analysis of qRT-PCR, the expression of related genes was calculated using the comparative CT method (2^−∆∆CT^). The Lineweaver–Burk double-reciprocal plots were constructed using GraphPad 9.4.1. The IC50 values were calculated using the Least squares regression methods. Statistical differences between groups were analyzed with One-way ANOVA. The *, **, and *** represent significant differences with *p* < 0.05, < 0.01, and < 0.001.

### Ethical approval

A total of 75 healthy yellow catfish were procured from a local market with an average body weight of 22.6 ± 1.2 g and a body length of 11.0 ± 0.2 cm. These fish underwent a 24-h fasting period before sampling. Subsequently, the fish were euthanized with CO_2_ asphyxiation. The experiment was conducted in accordance with relevant guidelines and legislations. The study complied with ARRIVE guidelines. The School of Life and Sciences at Jiangsu University examined and authorized the experiments involving animal participants.

### Supplementary Information

Supplementary Information 1.

Supplementary Information 2.

## Data Availability

The datasets generated during and/or analysed during the current study are available from the corresponding author on reasonable request.
