# Supplementary material for: Kinetic properties of glucose 6-phosphate dehydrogenase and inhibition effects of several metal ions on enzymatic activity in vitro and cells
Source: Sci Rep. 2024 Mar 9;14:5806. doi: 10.1038/s41598-024-56503-6 (PMC10924972; doi:10.1038/s41598-024-56503-6)
Supplement: Supplementary file 1 — Supplementary Information 1. [file 41598_2024_56503_MOESM1_ESM.pdf]

Kinetic properties of glucose 6-phosphate dehydrogenase (G6PD) and inhibition effects of several metal ions on enzymatic activity *in vitro* and cells

Lindan Sun[a], Binbin Sun[a], Yulei Zhang[b], Keping Chen[a*]

[a]School of Food and Biological Engineering; Institute of Life Sciences; Jiangsu University, Zhenjiang 212013, China.

[b]Guangdong South China Sea Key Laboratory of Aquaculture for Aquatic Economic Animals, Guangdong Ocean University, Zhanjiang 524088, China.

[*]Correspondence:

Prof. Dr. Keping Chen, E-mail address: 2212217022@stmail.ujs.edu.cn; School of Food and Biological Engineering; Institute of Life Sciences, Jiangsu University, Zhenjiang, Jiangsu, 212000, China.

Supplementary files 1: Description of original SDS-PAGE image of G6PD

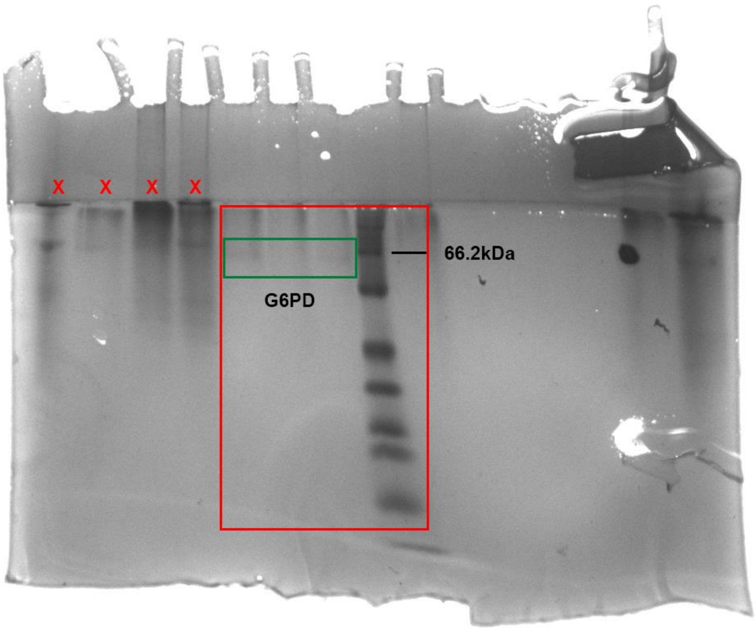

Caption: Figure 1b in the manuscript was captured from the Red Box of the SDS-PAGE raw image of G6PD and increased the contrast to clarify the band. Lane 1 shows a single band of G6PD (Green box) and was used for subsequent enzyme activity assays. The red "X" letter indicates that Figure 1b does not include these lanes.

Supplementary files 2: SDS-PAGE raw image of G6PD

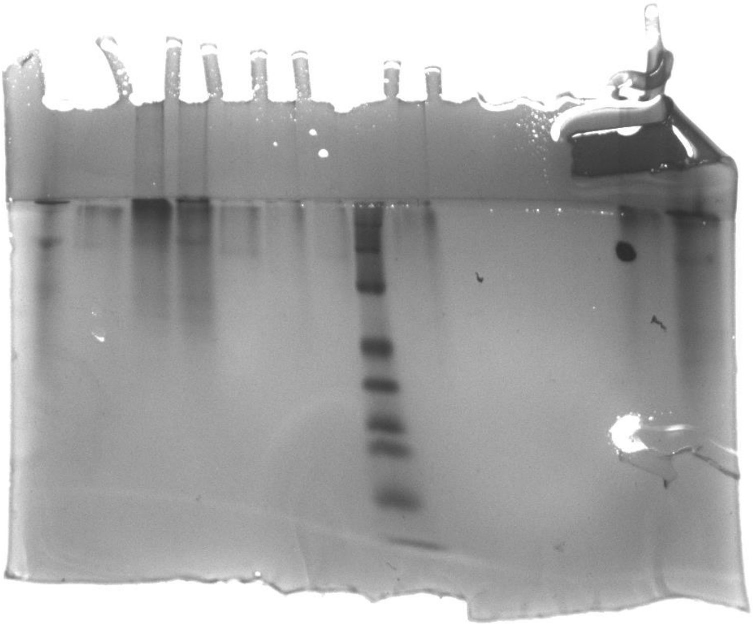

Caption: The original image was obtained by gel imaging instrument (GenoSens 1850).
